# Exploring neuropsychiatric symptoms in Friedreich ataxia

**DOI:** 10.1038/s41598-024-80258-9

**Published:** 2024-11-23

**Authors:** Simona Karamazovova, Lucie Stovickova, Dylan J. Jester, Veronika Matuskova, Jaroslava Paulasova-Schwabova, Michaela Kuzmiak, Alena Zumrova, Ross Andel, Martin Vyhnalek

**Affiliations:** 1 Center of Hereditary Ataxias, Department of Neurology, 2nd Faculty of Medicine and Motol University Hospital, Charles University, Prague, Czech Republic; 2 Center of Hereditary Ataxias, Department of Pediatric Neurology, 2nd Faculty of Medicine and Motol University Hospital, Charles University, Prague, Czech Republic; 3 Women’s Operational Military Exposure Network Center of Excellence (WOMEN CoE), VA Palo Alto Health Care System, Palo Alto, CA USA; 4 Edson College of Nursing and Health Innovation, Arizona State University, Phoenix, AZ USA

**Keywords:** Friedreich ataxia, Neuropsychiatric symptoms, Cerebellum, MBI-C, Neuroscience, Diseases of the nervous system, Emotion, Motivation

## Abstract

Neuropsychiatric symptoms (NPS) are common in hereditary ataxias as a part of the cerebellar cognitive affective syndrome. In Friedreich ataxia (FRDA), one of the most common hereditary ataxias, depressive symptoms were previously reported, but little is known about other NPS. We aimed to study the presence and severity of a broad range of NPS in individuals with FRDA and assess the relationship between the NPS and the disease severity, cognition, and quality of life and to examine the concordance between the NPS reported by the patients and by their informants. Mild Behavioral Impairment Checklist (MBI-C), a questionnaire designed for screening NPS in the early stages of neurodegenerative diseases, was administered to informants of individuals with FRDA and healthy controls and to people with FRDA themselves. Compared to healthy controls, patients with FRDA scored significantly higher in the total MBI-C score, emotion dysregulation domain (corresponding to depression and anxiety), and decreased motivation domain. When assessed by caregiver, the total MBI-C score and several NPS domains correlated with activities of daily living. Only psychotic symptoms were related to ataxia severity and general cognition. When endorsed by patients, only the relation between few MBI-C domains and quality of life was observed. We found slight to moderate agreement between informant-rated and patient-rated scores. NPS, particularly emotion dysregulation and decreased motivation, are common and clinically relevant in FRDA and should receive more attention due to their potential impact on quality of life and the possibility of therapeutic intervention.

**Supplementary Information:**

The online version contains supplementary material available at 10.1038/s41598-024-80258-9.

## Introduction

The neurophysiological role of the cerebellum goes well beyond motor control, involving a wide range of cognitive and emotional processes. Cognitive and neuropsychiatric impairment is thus a common result of various cerebellar disorders. The impairment forms a typical set of symptoms, the so-called cerebellar cognitive affective syndrome (CCAS)^1,2^. With respect to cognition, the CCAS is comprised of deficits in the executive, visuospatial, and language functions. The CCAS also includes an affective component that manifests as neuropsychiatric symptoms (NPS) of altered mood regulation, personality change, disinhibition and impulsive behavior, irritability, blunted affect, and even psychotic symptoms. These NPS have been described in cerebellar diseases of various etiology, including vascular, neurodegenerative, or tumors^1,3–5^.

Hereditary ataxias are a heterogeneous group of neurodegenerative diseases affecting the cerebellum and its connections. One of the most common hereditary ataxias worldwide is Friedreich ataxia (FRDA). It is an autosomal recessive disorder caused by a repeat expansion (or less commonly a point mutation) in the frataxin (*FXN*) gene leading to mitochondrial dysfunction. The disease’s neuropathology involves mainly the cerebellum’s dentate nucleus, the spinocerebellar and corticospinal tracts, and the posterior columns of the spinal cord. The classic clinical phenotype includes progressive gait and limb ataxia, pyramidal signs, peripheral neuropathy, dysarthria, eye movement abnormalities, scoliosis, foot deformity, cardiomyopathy, and diabetes mellitus^6^.

The prominent involvement of the cerebellum and its tracts in FRDA suggests the presence of the CCAS, and several studies have indicated the presence of cognitive and affective symptomatology, although there is not a complete consensus in this regard and further research on CCAS in FRDA is needed^7–9^. Furthermore, little attention has been paid to the full scope of the NPS in the existing studies. In other neurodegenerative diseases, including spinocerebellar ataxias, various NPS have been shown to significantly impact patients’ quality of life and activities of daily living^10–13^. In addition, unlike cerebellar motor impairment, which is largely irreversible, NPS such as depression, anxiety or psychotic symptoms may respond to pharmacotherapy or psychotherapy, as documented in several case studies and in line with the recommendations of the Clinical management guidelines for Friedreich ataxia^14–17^. Therefore, further research in this area is desirable.

Previous research found the presence of depression in individuals with FRDA, although the results have not been consistent across studies^18–22^. Only a few studies so far have examined other neuropsychiatric domains such as anxiety, agitation, or psychosis^8,21,23,24^. For example, several studies addressed anxiety in patients with FRDA but did not find a significant difference in anxiety levels compared to healthy controls^21,23^. To the best of our knowledge, only three studies focused on broader range of NPS, with varying methods developed mostly for psychiatric disorders^8,23,24^. One of the studies reported increased irritability, poor impulse control, and blunted affect in half of their cohort^8^. Few case reports documented psychotic symptoms such as auditory, visual, and somatic hallucinations and paranoid delusions, usually later in the disease course^14,16,25^.

To date, no instrument for assessing a broad range of NPS in ataxias has been recommended, and until now, domain-specific instruments relying on self-report such as the Beck Depression Inventory (BDI)^7,18,20,26^ or the Hospital Anxiety and Depression Scale (HADS)^21^ have been used in FRDA studies. Two studies also used structured or semi-structured interviews for DSM-IV disorders^23,24^. These methodological differences might account for some inconsistencies in the results, and some methods may not be sufficiently sensitive in patients with FRDA. Another potential pitfall is that many commonly used instruments contain items that can be influenced by motor impairment (e.g., unsteadiness, tremor, reduction of physical movement).

The standard instrument for studying NPS in neurodegenerative diseases is the Neuropsychiatric Inventory (NPI)^27^ or its shortened version, the Neuropsychiatric Inventory Questionnaire (NPI-Q)^28^. However, the NPI was designed for evaluating major psychopathology in patients with dementia and may not be sensitive enough for individuals with FRDA, in whom the NPS are presumed to be subtle and who usually present with only minor cognitive impairment^21–23^.

Recently, a new instrument called the Mild Behavioral Impairment Checklist (MBI-C) has been developed for assessing neuropsychiatric impairment in the early stages of neurodegenerative diseases^29^. The instrument is related to the concept of Mild Behavioral Impairment (MBI) which is defined as a new-onset and persisting change in behavior, mood or personality causing at least minimal impairment in a non-demented person over 50 years of age^30^. The questionnaire covers a broad spectrum of NPS divided into five domains (i.e., decreased motivation, emotional dysregulation, impulse dyscontrol, social inappropriateness, and abnormal perception or thought content). It aims at detecting the earliest signs of neuropsychiatric impairment in non-demented patients. MBI-C was initially developed to capture NPS in preclinical and prodromal Alzheimer disease and other dementing conditions, but its utility was also observed in motor disorders such as Parkinson disease or amyotrophic lateral sclerosis and other motor neuron diseases^31–35^. Since FRDA is a neurodegenerative disorder characterized by predominant motor involvement and relatively minor cognitive impairment that typically does not reach the level of dementia, the MBI framework could be well suited for detecting NPS in people with FRDA. Another advantage of the questionnaire is that it does not contain items that could be influenced by motor impairment and thus be misinterpreted. Moreover, the MBI-C assesses symptoms over a 6-month time frame, which helps to capture more persistent behavioral changes, whereas other commonly used scales have much shorter timeframes and may include more transient symptoms. The MBI-C has been translated to several languages and is freely available^36^. The questionnaire is self-administered and can be completed by an informant as well as by the patient, the former being the most common approach.

Previous research on neurodegenerative diseases, including spinocerebellar ataxias (SCA) or Parkinson disease has shown significant discrepancies in the spectrum and prevalence of NPS when evaluated by patients and their informants, such as family members and close friends. Agreement between patients and informants was generally low. Namely, patients tended to report depression, hallucinations, and sleep disturbances more often, while informants emphasized symptoms of irritability and agitation^5,37,38^. These differences may be due to different perspectives or due to patients´ anosognosia for certain symptoms (i.e., lack of insight). Informant input can be particularly valuable in assessing the full impact of NPS but, to date, no study has examined it in patients with FRDA.

Using the MBI-C, we aimed to (1) establish its efficacy in detecting the presence and severity of a wide range of NPS in FRDA, (2) analyze the agreement between the NPS reported by the patients themselves with those endorsed by their informants, and (3) examine the relationship between these symptoms and the ataxia severity, general cognition and quality of life. Based on previous findings, we hypothesized that the MBI-C could detect neuropsychiatric impairment in FRDA. This impairment would be more severe in individuals with FRDA than in healthy controls, not only in the emotional dysregulation domain but in other NPS domains as well. We also hypothesized that patients would endorse more NPS in the emotional dysregulation domain whereas their informants would report significantly more NPS in other domains. Finally, we hypothesized that the frequency and severity of the NPS would correlate with the severity of ataxia, impairment of activities of daily living, and quality of life.

## Methods

### Participants

We recruited patients with FRDA from the Center of Hereditary Ataxias in Prague, Czech Republic, a member of the European Reference Network for Rare Neurological Diseases (ERN-RND) and the European Friedreich ataxia Consortium for Translational Studies (EFACTS). All patients had a genetically confirmed diagnosis of FRDA and were clinically symptomatic individuals over the age of 16. In particular, all but one patient were homozygous for GAA expansions in the *FXN* gene, and one individual was a compound heterozygote for a GAA expansion and a point mutation.

Exclusion criteria were other brain diseases potentially causing NPS (stroke, other neurodegenerative diseases such as Parkinson or Alzheimer disease, craniocerebral trauma, etc.), and history of substance abuse. Patients with significant pathology on brain MRI besides the brainstem and cerebellum atrophy (such as significant vascular changes, tumors, etc.) and patients with acutely exacerbated general medical conditions such as non-compensated diabetes mellitus, renal or cardiac failure and other diseases potentially influencing cognition or causing NPS were not included. We also excluded participants who did not complete two or more items on the MBI-C questionnaire.

The control group consisted of age- and education-matched healthy volunteers (HV), based on availability, recruited via advertising on social networks, or among the researchers’ personal acquaintances. HV reported no significant cognitive complaints, history of any brain disease, substance abuse, or a general medical condition potentially causing NPS.

All participants gave their written informed consent and the study was conducted with the approval of the local ethics committee of Motol University Hospital and in accordance with the Helsinki Declaration.

### Procedures

All patients with FRDA underwent detailed neurological examination in Czech, including the Scale for the Assessment and Rating of Ataxia (SARA)^39^, the Montreal Cognitive Assessment (MoCA)^40^, the self-reported evaluation of the Activities of Daily Living (ADL)^41^, and the self-reported visual analogue scale of health-related quality of life (EQ-5D VAS)^42^. Healthy volunteers underwent a clinical interview focused on their potential cognitive complaints and medical history.

Three patients were unable to complete the trail-making, cube-drawing, and clock-drawing tasks in the MoCA due to severe motor impairment. To overcome this obstacle, we subtracted these tasks from the maximum score, counted the percentage of correct items completed by the patient, and calculated a score corresponding to the same percentage of the maximum score. We labeled the new score MoCA corrected, as shown in Tables 1, 2 and 3.

**Table 1:** Demographic and clinical characteristics of the sample, MBI-C results.

Characteristics	FRDA *n* = 33 M ± SD (range)	HV *n* = 49 M ± SD (range)
Age	37.39 ± 13.33 (16–72)	36.90 ± 12.66 (20–77)
Female, n (%)	14 (42.42%)	32 (65.31%)
Education, years	14.52 ± 3.42 (9–22)	15.86 ± 2.83 (12–24)
Disease duration, years	23.36 ± 10.54 (2–45)	
Age of onset	16.39 ± 10.14 (1–54)	
SARA (0–40)	23.90 ± 8.56 (8–40)	
ADL (0–36)	16.55 ± 6,25 (2–27)	
EQ-5D VAS (0-100)	58.47 ± 21.21 (24–97)	
MoCA corrected (0–30)	26.50 ± 2.57 (19–30)	
MBI-C informant-rated (obtained from 33 FRDA and 49 HV)		
Total (0-102) ≥ 1 (%) ≥ 7 (%)	6.33 ± 8.36 (0–32)** 66.7% 36.4%**	**2.10 ± 2.92 (0–12)** 53.1% **8.2%**
Decreased motivation (0–18) ≥ 1 (%)	1.33 ± 3.03 (0–14)* 24.2%	**0.35 ± 0.90 (0–4)** 16.3%
Emotional dysregulation (0–18) ≥ 1 (%)	2.94 ± 3.85 (0–12)** 57.6%*	**0.82 ± 1.60 (0–6)** **28.6%**
Impulse dyscontrol (0–36) ≥ 1 (%)	1.61 ± 2.47 (0–9) 45.5%	0.76 ± 1.52 (0–8) 32.7%
Social inappropriateness (0–15) ≥ 1 (%)	0.15 ± 0.44 (0–2) 12.1%	0.06 ± 0.32 (0–2) 4.1%
Abnormal perception/thought (0–15) ≥ 1 (%)	0.30 ± 1.1 (0–6) 12.1%	0.12 ± 0.44 (0–2) 8.2%
MBI-C patient-rated (obtained from 23 FRDA)		
Total (0-102) ≥ 1 (%) ≥ 7 (%)	6.34 ± 8.28 (0–39) 76.8% 43.48%	
Decreased motivation (0–18) ≥ 1 (%)	1.47 ± 3.23 (0–17) 36.3%	
Emotional dysregulation (0–18) ≥ 1 (%)	3.13 ± 3.91 (0–15) 66.7%	
Impulse dyscontrol (0–36) ≥ 1 (%)	1.00 ± 1.70 (0–6) 33.3%	
Social inappropriateness (0–15) ≥ 1 (%)	0.31 ± 0.69 (0–3) 21.2%	
Abnormal perception/thought (0–15) ≥ 1 (%)	0.41 ± 1.19 (0–6) 18.2%	

* difference from HV group at *p* < .05, ** difference from HV group at *p* < .01.

The results of the MBI-C questionnaire are presented as mean ± SD (range); in addition, the relative number of participants scoring ≥ 1 on the total and domain scores and ≥ 7 on the total score are presented.

**Table 2:** Correlations with informant-rated MBI-C (correlation coefficient, p-value).

	MBI-C total	Decreased motivation	Emotional dysregulation	Impulse dyscontrol	Social inappropriateness	Abnormal perception/thought
Disease duration	0.11, *p* = .39	-0.12, *p* = .39	0.13, *p* = .33	0.05, *p* = .73	**0.38**, ***p*** = .01*	0.06, *p* = .70
SARA	0.09, *p* = .50	0.09, *p* = .52	0.14, *p* = .28	0.04, *p* = .75	0.15, *p* = .30	**0.33**, *p* = .02*
ADL	**0.27**, ***p*** = .04*	**0.30**, ***p*** = .04*	**0.32**, ***p*** = .01*	0.19, *p* = .17	0.13, *p* = .39	**0.36**, p = .01*
EQ-5D VAS	-0.21, *p* = .12	-0.05, *p* = .75	-0.13, *p* = .35	-0.32, *p* = .15	-0.18, *p* = .23	-0.06, *p* = .71
MoCA corrected	-0.12, *p* = .37	-0.07, *p* = .63	-0.12, *p* = .40	-0.11, *p* = .46	-026, *p* = .09	**-0.33**, ***p*** = .03*

Significant values are in bold.

**Table 3:** Correlations with self-rated MBI-C (correlation coefficient, p-value).

	MBI-C total	Decreased motivation	Emotional dysregulation	Impulse dyscontrol	Social inappropriateness	Abnormal perception/thought
Disease duration	-0.06, *p* = .67	-0.16, *p* = .24	0.01, *p* = .95	-0.12, *p* = .41	-0.08, *p* = .57	-0.02, *p* = .90
SARA	0.00, *p* = .99	0.02, *p* = .90	0.06, *p* = .67	-0.01, *p* = .95	-0.21, *p* = .15	-0.07, *p* = .65
ADL	0.06, *p* = .66	0.08, *p* = .57	0.15, *p* = .26	0.01, *p* = .94	-0.15, *p* = .30	-0.06, *p* = .69
EQ-5D VAS	**-0.31**, *p* = .02*	**-0.29**, *p* = .04*	**-0.36**, ***p*** = .01*	-0.14, *p* = .35	-0.13, *p* = .41	0.12, *p* = .42
MoCA corrected	-0.07, *p* = .61	0.02, *p* = .92	-0.01, *p* = .96	-0.20, *p* = .19	-0.15, *p* = .33	-0.13, *p* = .39

Significant values are in bold.

The Czech version of the Mild Behavioral Impairment Checklist (MBI-C)^43^ was completed by the patients’ and healthy volunteers’ proxies (relatives, close friends, or formal caregivers). A subset of individuals with FRDA also completed the patient version of the MBI-C themselves.

The MBI-C consists of 34 items organized into five domains, i.e., (1) decreased motivation, (2) emotional dysregulation, (3) impulse dyscontrol, (4) social inappropriateness, and (5) abnormal perception or thought content. Each item depicts a particular symptom and requires a “yes” or “no” answer. In case of a positive answer, an assessment of the symptom severity on a scale from 1 (mild) to 3 (severe) is required. The symptoms should persist for at least 6 months and represent an impactful change from previous behavior. Domain scores are calculated as a sum of the corresponding item severity ratings, resulting in decreased motivation score (0–18), emotional dysregulation score (0–18), impulse dyscontrol score (0–36), social inappropriateness score (0–15), and abnormal perception or thought content score (0–15). A total score (0-102) is calculated as a sum of the domain scores.

### Statistical analysis

Descriptive statistics were reported for the study variables. A two-sample t-test was used to compare differences in age and education, and a chi-squared test was used to compare differences in gender proportions.

To assess the difference in total MBI-C scores and MBI-C domain scores between FRDA and HV, we used a negative binomial regression model with sex as covariate. To further address the effect of age on NPS, we performed negative binomial regression models with FRDA status as the independent variable, MBI-C components as the various dependent variables, and sex and age as covariates.

Negative binomial regression was chosen over linear regression due to the skewed distributions of the MBI-C total and domain scores, which presented similarly to count outcomes. Beta coefficients were exponentiated and reported as incidence rate ratios (IRR) with 95% confidence intervals and p-values.

We also calculated the percentage of patients and controls who scored more than zero in the total score and each domain score and used the chi-square test to compare the frequencies between the groups. We applied kappa coefficients for inter-rater reliability to analyze the concordance between the informant-rated and patient-rated scores.

Wilcoxon signed-rank tests were used to estimate the differences in domain and total MBI-C scores between proxy- and self-administered scales. In the FRDA group, we used the Kendall rank correlation coefficient to examine the relationship between both informant-rated and patient-rated MBI-C scores and the disease duration, SARA, ADL, EQ-5D VAS, and MoCA corrected. All analyses were performed in RStudio^44^ with a p-threshold for statistical significance (p-value) set at a two-tailed 0.05 level.

## Results

Thirty-three patients with FRDA and 49 HV were enrolled and their proxies completed the informant-rated MBI-C. Twenty three of those 33 patients also completed the patient-rated version of MBI-C. The basic demographic and clinical characteristics and MBI-C scores of both groups are summarized in Table 1. The groups did not differ in age or education, but the HV group had a higher proportion of women. Four patients had late-onset FRDA (age of onset > 25 years).

Eleven patients (33.3%) were currently taking antidepressants, most commonly sertraline. One patient had a history of bipolar disorder diagnosed at the age of 24, nine years after the onset of neurological symptoms linked to FRDA and was stable on valproate and sertraline during data collection. The remaining patients on antidepressant treatment exhibited depressive symptomatology during the clinically established neurological stage, and in these cases, treatment was initiated by the neurologist.

The MBI-C questionnaire was well accepted; only one patient (not included in the 33 patients reported above) was excluded due to missing data on the informant-rated questionnaire. We did not encounter any other issues with compliance.

In the FRDA group, the informant-rated version of the MBI-C was most commonly completed by a parent (15/33, 45%), followed by a partner (9/33, 27%), a formal caregiver (4/33, 12%), a sibling (2/33, 6%), and a friend (1/33, 3%). In two cases this information was missing. In HV, the questionnaire was most often completed by a partner (27/49, 55%), followed by a child (9/49, 18%), a friend (7/49, 14%), a parent (4/49, 8%), and a sibling (2/49, 4%).

In the informant-rated version of the MBI-C questionnaire, the individuals with FRDA had significantly higher total scores than HV (IRR = 3.11 [1.55, 6.41], *p* = .002). Patients also scored significantly higher in the decreased motivation (IRR = 4.02 [1.03, 17.38], *p* = .048) and emotional dysregulation domains (IRR = 3.71 [1.63, 8.77], *p* = .003). Differences in other domains were not significant, although the impulse dyscontrol score approached significance (IRR = 2.32 [0.95, 5.90], *p* = .056). In the fully adjusted model with sex and age as covariates, the age was associated only with MBI impulse dyscontrol and MBI social inappropriateness, and the main effects of FRDA did not change markedly from the sex-adjusted models (see tables S1 and S2 in the Supplement).

Overall, 66.7% (22/33 individuals) of the FRDA group had a total MBI-C score ≥ 1, compared to 53.1% (26/49 individuals) of the HV group. The percentage of participants scoring > 0 in each particular domain is shown in Table 1. In particular, the psychotic symptoms were endorsed in 12% of patients and these were among the most impaired in terms of SARA (25.5–40) and ADL (19–27). We found a significant difference in the frequency only in the emotional dysregulation domain (*p* = .009).

Twelve patients in the FRDA group (36.4%) exbihited high severity NPS (MBI-C total score ≥ 7), compared to 4 participants in the HV group (8.2%), which constitutes a significant difference (*p* = .002). In the subset of patients who completed the patient-rated version of MBI-C, ten patients (43.48%) reported high-severity symptoms.

Concerning the agreement between informant-rated and patient-rated scores, we found slight to fair agreement for most subdomains, and moderate agreement for decreased motivation (total score: κ = 0.287, *p* = .0945; emotion dysregulation: κ = 0.261, *p* = .132; abnormal perception or thought: κ = 0.294, *p* = .0869; impulse dyscontrol: κ = 0.108, *p* = .529; decreased motivation: κ = 0.429, *p* = .0114; social inappropriateness: κ = 0.243, *p* = .146). Only decreased motivation reached statistical significance (i.e., informants and patients agreed at a rate much better than chance). We did not find differences in any total or domain scores between the proxy- and self-administered scales (all *p* ≥ .13). In other words, patients or informants did not report higher or lower values than each other - they were statistically equivalent. For a detailed representation of individual concordance between informants and patients, see figure S1 in the Supplement.

Analyzing the correlations between the NPS and disease duration, ataxia severity, quality of life, and general cognition in patients with FRDA, we found that in the informant-rated MBI-C (Table 2), disease duration positively correlated with the social inappropriateness domain (τ = 0.38, *p* = .01), SARA positively correlated with abnormal perception or thought content (τ = 0.33, *p* = .02), and ADL positively correlated with the total MBI-C score (τ = 0.27, *p* = .04), decreased motivation (τ = 0.30, *p* = .04), emotional dysregulation (τ = 0.32, *p* = .01), and abnormal perception or thought content (τ = 0.36, *p* = .02). MoCA corrected correlated negatively with abnormal perception or thought content (τ=-0.33, *p* = .03).

In the patient-rated version of the MBI-C (Table 3), we found negative correlations between the EQ-5D VAS and the total score (τ=-0.31, *p* = .02), decreased motivation (τ=-0.29, *p* = .04), and emotional dysregulation (τ=-0.36, *p* = .008).

## Discussion

In this study, we focused on assessing the most common domains of NPS in FRDA. We have demonstrated that the MBI-C questionnaire could detect neuropsychiatric impairment in FRDA. The NPS, particularly decreased motivation and emotion dysregulation, were present in individuals with FRDA and were partially related to activities of daily living, ataxia severity, general cognition, disease duration, and quality of life.

Our work extends the previous research on NPS in FRDA, which focused mainly on depressive symptoms^18–20,45^. We confirmed and further expanded these findings by studying other NPS domains, and found a significant difference in motivation compared to healthy individuals in the informant-rated MBI-C. We did not find a difference in the other domains (impulse dyscontrol, social inappropriateness, and psychotic symptoms), although the score in impulse dyscontrol approached significance.

A substantial proportion of individuals with FRDA in our cohort were taking antidepressants, which could have substantially influenced NPS and, consequently, the MBI-C score, potentially underreporting depressive symptoms in the FRDA population.

It is also worth noting that although psychotic symptoms were rare, which is consistent with previous research^14–16,25,46^, they were present in over 12% of patients. Identifying these individuals is important in clinical care in order to address the psychotic symptoms, which may constitute an additional burden for patients and caregivers. In several published case studies, antipsychotics such as quetiapine, aripiprazole, and risperidone were reported as efficacious in treating psychotic symptoms in patients with FRDA and based on this evidence, the current Clinical management guidelines for Friedreich ataxia recommend the use of antipsychotics for confirmed episodes of psychosis^14–17^.

Although the MBI-C questionnaire was originally developed for capturing NPS in the preclinical stages of dementing conditions such as Alzheimer disease, several studies have used it in movement disorders, namely Parkinson disease (PD)^32–34^. While the people with PD in these studies were generally older than our patients with FRDA, their MBI-C scores were often very similar. Mean total MBI-C scores in PD ranged from 5.19 to 6.1 (compared to 6.33 in our cohort). Individuals with FRDA scored higher than those with PD on emotional dysregulation and lower on social inappropriateness. Scores on motivation, impulsivity, and psychotic symptoms were comparable among the two groups. Psychosis is considered to be common in PD^47^; the similar score in psychotic symptoms (ranging from 0.3 to 0.86 in PD compared to 0.3 in FRDA) is thus particularly intriguing and underscores the need for screening and management of these symptoms in FRDA.

While the agreement between informant-rated and patient-rated questionnaires is only slight to moderate according to the kappa coefficients, we did not find any systematic differences between informants and patients in assessing the particular domains as we hypothesized and as was demonstrated in studies with SCA, Parkinson disease, dementia or mild cognitive impairment^5,37,38,48^. However, the lack of such differences might be due to the small sample size, milder severity of NPS in people with FRDA compared to other neurodegenerative diseases, and/or the psychometric properties of the scales themselves.

The correlations between informant-rated and patient-rated MBI-C scores and SARA, ADL, EQ-5D VAS, and MoCA corrected showed a noteworthy pattern. While the informant-rated total score and emotional dysregulation and decreased motivation scores correlated with ADL, the same scores rated by the patient correlated with the quality of life. None of these were associated with cognitive impairment or ataxia severity. These results suggest that informant-, but not patient-rated NPS are related to the level of functioning in daily activities, but only patient-rated NPS are related to the subjectively perceived quality of life. Literature suggests that NPS have a significant impact on the quality of life in many neurodegenerative diseases, including cerebellar disorders^10,11,49^. Based on our results, we could thus hypothesize a similar effect in patients with FRDA. On the other hand, inverse causality is also possible, i.e. people with lower quality of life due to motor or other impairments may become depressed or less motivated as a consequence. Further research is necessary to clarify this relationship. The correlation between the informant-rated scores and ADL may indicate a more objective view and underscores the importance of considering the caregivers’ insights that could help to fully evaluate the impact of the NPS.

Informant-rated abnormal perception or thought content was the only domain that showed a positive correlation not only with ADL but also with SARA and a negative correlation with general cognition. These results must be treated with caution, given the small number of patients exhibiting psychotic symptoms. However, the relationship with SARA and ADL is consistent with previous literature showing that psychotic symptoms tend to occur in patients with more severe motor impairment in the later stages of the disease, as was the case in our study^14–16,25,46^.

As for the lack of correlation between general cognition and most of the NPS domains, a possible reason may be the insufficient sensitivity of the MoCA in detecting cerebellar cognitive impairment. Future studies could evaluate the correlation of the NPS with cognition measured by a comprehensive neuropsychological battery.

The NPS in FRDA may either result from the process of cerebellar neurodegeneration or be a consequence of living with an untreatable, highly disabling disease, or a combination of both. The correlations with ADL may point toward the possibility that NPS in FRDA arise as a reaction to the increasing burden of living with the progressive disease. In this regard, a comparison with SCA, another subgroup of hereditary ataxias with a different cerebellar and extra-cerebellar degeneration pattern, is particularly interesting. Most studies on NPS in SCA do not find a linear relationship between measures of disease progression and NPS^49–52^. In fact, patients with SCA usually have less severe motor disability yet more pronounced NPS than patients with FRDA^49^. We can postulate that in SCA, neurodegeneration is the primary factor contributing to the onset of NPS, whereas in FRDA, the link between NPS and the increasing disability appears more prominent. However, further research, particularly involving neuroimaging techniques with a focus on volumetric analysis of cerebellar structures relevant to neuropsychiatric functions, is essential to resolve this complex issue.

The main strength of this paper is that it is the first study to assess NPS in FRDA comprehensively using a standardized questionnaire involving reports from both the patients and their informants. We demonstrated impairment not only in the previously reported affective regulation, but also in motivation. Additionally, our study shows that the MBI-C may be a suitable tool for further research on NPS in FRDA, as it is well accepted by patients and caregivers and is not influenced by motor impairment.

There are also some limitations worth noting. The MBI-C was developed for patients over the age of 50 years, and has not been validated in younger patients. However, it is a comprehensive questionnaire designed for patients with neurodegenerative disorders and has been used previously in other movement disorders^32–35^. Another limitation is the relatively small number of patients in our study, although FRDA is a rare disease, and our cohort represents the majority of diagnosed patients in the Czech Republic. Concerning all the aforementioned correlations, it is necessary to point out that we did not correct for multiple comparisons. This is an exploratory study on a small cohort of patients; hence the results are inherently biased towards Type II error, which correction for multiple comparisons could exacerbate. We acknowledge that our pilot results need to be confirmed by future multicentric international research.

## Conclusion and future directions

In conclusion, our findings show that NPS are common in FRDA and manifest not only as depression and anxiety but also as decreased motivation. It is difficult to distinguish whether these NPS are due to the neurodegenerative process or to the burden of living with a progressive, devastating disease, but in any case they need to be considered in clinical care. Although we did not observe significant impairment in the other NPS domains, the presence of psychotic symptoms or impulsivity in individual patients may have adverse consequences on a personal level for both patients and caregivers and is therefore worth noting.

The NPS were unrelated to ataxia severity or general cognitive decline, except for caregiver endorsed psychotic symptoms.

In the absence of a specific validated instrument for hereditary ataxias, we propose the MBI-C as a useful questionnaire for studying NPS in individuals with FRDA since it is comprehensive yet simple to administer, unaffected by motor symptoms, and easily accessible in many languages. As only slight to moderate agreement between informant-rated and patient-rated scores was found and the results indicate that both versions convey different information, we suggest to administer both versions when examining NPS in people with FRDA.

Future studies that adopt the MBI-C could provide more robust results in larger cohorts of patients, employ neuroimaging methods to link the NPS to structural and functional changes in the cerebellum and other brain structures, and also examine the longitudinal course of neuropsychiatric impairment in FRDA.

## Electronic supplementary material

Below is the link to the electronic supplementary material.

Supplementary Material 1

## Data Availability

The datasets used and analyzed in this study are available upon reasonable request from the corresponding author.
