# Supplementary material for: Exploring neuropsychiatric symptoms in Friedreich ataxia
Source: Sci Rep. 2024 Nov 23;14:29076. doi: 10.1038/s41598-024-80258-9 (PMC11585572; doi:10.1038/s41598-024-80258-9)
Supplement: Supplementary file 1 — Supplementary Material 1 [file 41598_2024_80258_MOESM1_ESM.docx]

**Table S1 (original models) – Negative binomial regression with sex as a covariate**

|  | **MBI total** | | | **MBI emotional dysregulation** | | | **MBI abnormal perception or thought content** | | | **MBI impulse dyscontrol** | | | **MBI decreased motivation** | | | **MBI social inappropriateness** | | |
| --- | --- | --- | --- | --- | --- | --- | --- | --- | --- | --- | --- | --- | --- | --- | --- | --- | --- | --- |
| ***Predictors*** | ***Incidence Rate Ratios*** | ***CI*** | ***p*** | ***Incidence Rate Ratios*** | ***CI*** | ***p*** | ***Incidence Rate Ratios*** | ***CI*** | ***p*** | ***Incidence Rate Ratios*** | ***CI*** | ***p*** | ***Incidence Rate Ratios*** | ***CI*** | ***p*** | ***Incidence Rate Ratios*** | ***CI*** | ***p*** |
| **(Intercept)** | **1.80** | **0.98 – 3.51** | **0.083** | **0.68** | **0.32 – 1.51** | **0.347** | **0.07** | **0.01 – 0.37** | **0.003** | **0.63** | **0.27 – 1.54** | **0.269** | **0.38** | **0.13 – 1.29** | **0.134** | **0.05** | **0.01 – 0.33** | **0.001** |
| **FRDA [1]** | **3.11** | **1.55 – 6.41** | **0.002** | **3.71** | **1.63 – 8.77** | **0.003** | **2.48** | **0.47 – 14.77** | **0.311** | **2.32** | **0.95 – 5.90** | **0.056** | **4.02** | **1.03 – 17.38** | **0.048** | **2.64** | **0.40 – 21.15** | **0.290** |
| **sex [2]** | **1.28** | **0.63 – 2.59** | **0.502** | **1.34** | **0.57 – 3.11** | **0.509** | **2.35** | **0.40 – 14.82** | **0.354** | **1.27** | **0.51 – 3.19** | **0.587** | **0.83** | **0.19 – 3.28** | **0.786** | **1.17** | **0.17 – 8.97** | **0.867** |
| **Observations** | **82** | | | **82** | | | **82** | | | **82** | | | **82** | | | **82** | | |
| **R^2^Nagelkerke** | **0.173** | | | **0.184** | | | **0.091** | | | **0.072** | | | **0.117** | | | **0.053** | | |

**Table S2 – Negative binomial regression with sex and age as covariates**

|  | **MBI total** | | | **MBI emotional dysregulation** | | | **MBI abnormal perception or thought content** | | | **MBI impulse dyscontrol** | | | **MBI decreased motivation** | | | **MBI social inappropriateness** | | |
| --- | --- | --- | --- | --- | --- | --- | --- | --- | --- | --- | --- | --- | --- | --- | --- | --- | --- | --- |
| ***Predictors*** | ***Incidence Rate Ratios*** | ***CI*** | ***p*** | ***Incidence Rate Ratios*** | ***CI*** | ***p*** | ***Incidence Rate Ratios*** | ***CI*** | ***p*** | ***Incidence Rate Ratios*** | ***CI*** | ***p*** | ***Incidence Rate Ratios*** | ***CI*** | ***p*** | ***Incidence Rate Ratios*** | ***CI*** | ***p*** |
| **(Intercept)** | **2.61** | **0.80 – 8.95** | **0.098** | **1.81** | **0.48 – 7.23** | **0.394** | **0.21** | **0.01 – 4.92** | **0.286** | **0.19** | **0.03 – 1.08** | **0.019** | **1.71** | **0.21 – 17.48** | **0.637** | **0.00** | **0.00 – 0.09** | **<0.001** |
| **FRDA [1]** | **3.16** | **1.57 – 6.53** | **0.002** | **4.49** | **1.93 – 11.04** | **0.001** | **2.00** | **0.35 – 12.15** | **0.436** | **2.48** | **1.03 – 6.25** | **0.038** | **5.49** | **1.35 – 27.21** | **0.015** | **2.43** | **0.29 – 25.02** | **0.364** |
| **sex [2]** | **1.40** | **0.66 – 2.96** | **0.369** | **1.65** | **0.69 – 4.00** | **0.255** | **2.87** | **0.48 – 19.95** | **0.259** | **1.03** | **0.40 – 2.64** | **0.955** | **1.32** | **0.29 – 6.55** | **0.695** | **0.74** | **0.07 – 7.07** | **0.768** |
| **age** | **0.99** | **0.96 – 1.02** | **0.406** | **0.97** | **0.93 – 1.00** | **0.057** | **0.97** | **0.89 – 1.04** | **0.370** | **1.03** | **0.99 – 1.08** | **0.041** | **0.95** | **0.88 – 1.01** | **0.064** | **1.09** | **1.00 – 1.23** | **0.012** |
| **Observations** | **82** | | | **82** | | | **82** | | | **82** | | | **82** | | | **82** | | |
| **R^2^Nagelkerke** | **0.181** | | | **0.240** | | | **0.124** | | | **0.122** | | | **0.193** | | | **0.233** | | |

**Figure S1 – Agreement between patient- and informant-rated MBI-C scores for total and domain-specific scores in patients with FRDA.**


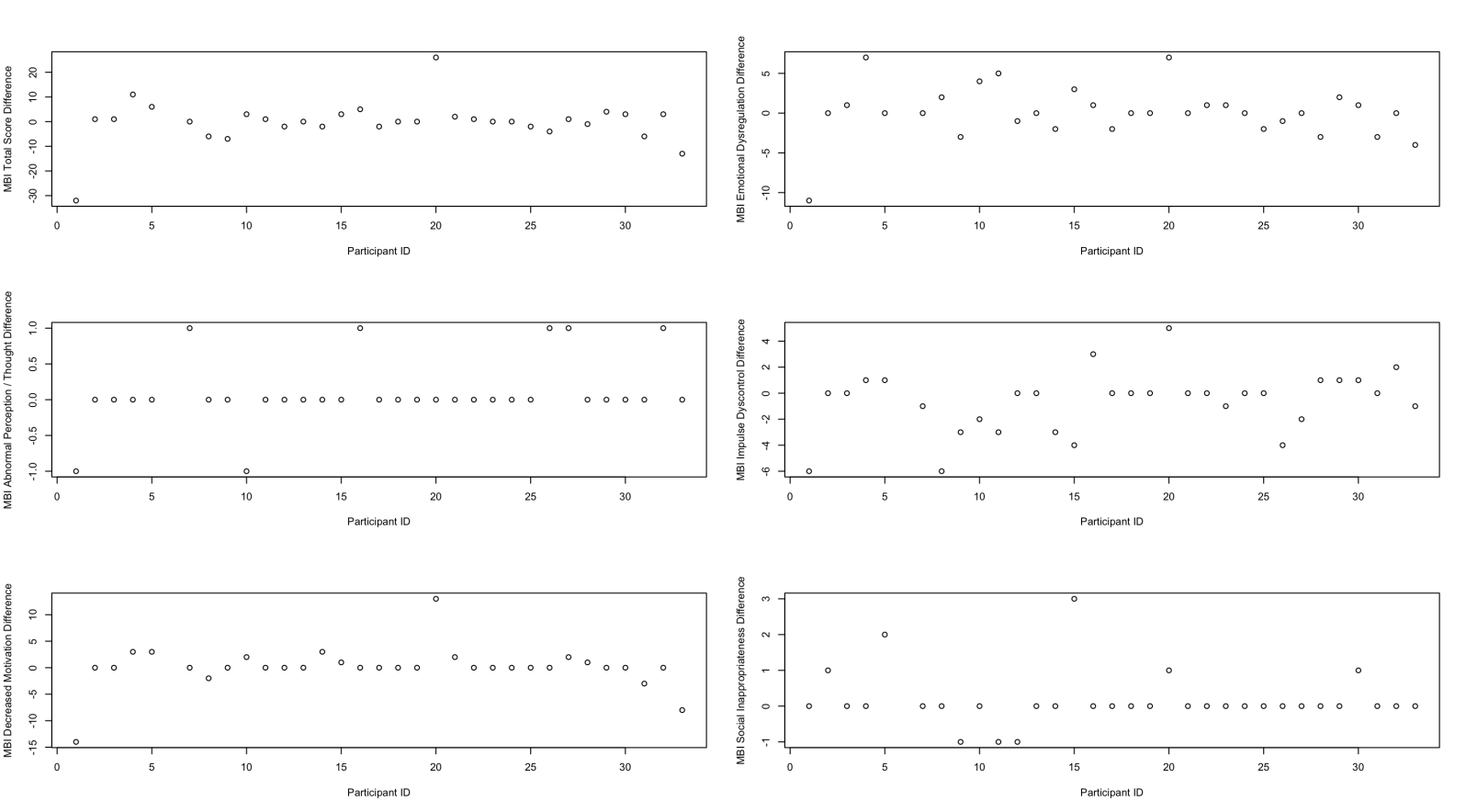
The Y-axis represents the difference between patient and informant ratings, where negative values indicate that the informant reported higher symptom severity than the patient, and positive values indicate that the patient reported higher symptom severity. A value of 0 indicates perfect agreement. The X-axis represents the participant ID.
